# The rat retrosplenial cortex as a link for frontal functions: A lesion analysis

**DOI:** 10.1016/j.bbr.2017.08.010

**Published:** 2017-09-29

**Authors:** Anna L. Powell, Andrew J.D. Nelson, Emma Hindley, Moira Davies, John P. Aggleton, Seralynne D. Vann

**Affiliations:** School of Psychology, Cardiff University, Tower Building, Park Place, Cardiff CF10 3AT, UK

**Keywords:** Cingulate cortex, Executive control, Extradimensional shift, Inhibition, Prelimbic cortex, Spatial memory, Strategy switch

## Abstract

Cohorts of rats with excitotoxic retrosplenial cortex lesions were tested on four behavioural tasks sensitive to dysfunctions in prelimbic cortex, anterior cingulate cortex, or both. In this way the study tested whether retrosplenial cortex has nonspatial functions that reflect its anatomical interactions with these frontal cortical areas. In Experiment 1, retrosplenial cortex lesions had no apparent effect on a set-shifting digging task that taxed intradimensional and extradimensional attention, as well as reversal learning. Likewise, retrosplenial cortex lesions did not impair a strategy shift task in an automated chamber, which involved switching from visual-based to response-based discriminations and, again, included a reversal (Experiment 2). Indeed, there was evidence that the retrosplenial lesions aided the initial switch to response-based selection. No lesion deficit was found on an automated cost-benefit task that pitted size of reward against effort to achieve that reward (Experiment 3). Finally, while retrosplenial cortex lesions affected matching-to-place task in a T-maze, the profile of deficits differed from that associated with prelimbic cortex damage (Experiment 4). When the task was switched to a nonmatching design, retrosplenial cortex lesions had no apparent effect on performance. The results from the four experiments show that many frontal tasks do not require the retrosplenial cortex, highlighting the specificity of their functional interactions. The results show how retrosplenial cortex lesions spare those learning tasks in which there is no mismatch between the internal and external representations used to guide behavioural choice. In addition, these experiments further highlight the importance of the retrosplenial cortex in solving tasks with a spatial component.

## Highlights

• Retrosplenial cortex lesions do not reproduce the pattern of effects of medial frontal damage.
• Retrosplenial cortex lesions spare tests of behavioural flexibility.
• Effort-based decision making does not require the retrosplenial cortex.
• Reveals specific conditions when nonspatial tasks engage retrosplenial cortex.

## 1 Introduction

Important clues to retrosplenial cortex (areas 29, 30) function come from its connectivity. This posterior cingulate region has dense interconnections with the anterior thalamic nuclei, as well as with hippocampal and parahippocampal regions [1], [2], [3], [4], [5], [6]. Reflecting these connections, the rat retrosplenial cortex contributes to spatial memory and navigation [7], [8], [9], [10], [11], [12]. Despite these findings, it is clear that the retrosplenial cortex has a very different role in spatial cognition from that of the hippocampus and anterior thalamic nuclei. Indeed, retrosplenial lesion deficits often only emerge when animals are required to switch flexibly between different spatial strategies or competing cue types [13], [14], [15]. Such evidence potentially points to a broader role for the retrosplenial cortex in cognition beyond the spatial domain. A consideration of its other anatomical connections is consistent with this proposal. Retrosplenial cortex is also heavily interconnected with the anterior cingulate cortex [16], [17], [18] which, in turn, is closely connected with other frontal areas, including prelimbic cortex [19]. Retrosplenial cortex is also indirectly linked with prelimbic cortex via its dense anterior thalamic and medial thalamic connections [16], [19]. Yet, the functional importance of these retrosplenial-frontal connections remains poorly understood.

The present study, therefore, addressed the question of whether retrosplenial cortex contributes to functions associated with the rodent anterior cingulate and prelimbic cortices. Preliminary support for this view comes from the finding that retrosplenial cortex lesions disrupt a rodent analogue of the Stroop task [20], which is also sensitive to medial frontal cortex lesions [21]. In addition, retrosplenial cortex lesions can disrupt recency judgements [22], an ability closely associated with medial frontal cortex function in rats [23], [24], [25] as well as crossmodal recognition memory [26], [27].

To provide a more comprehensive answer, rats with retrosplenial cortex lesions were tested on a series of four behavioural tasks thought to rely on either the prelimbic cortex, anterior cingulate cortex, or both cortical areas. In Experiment 1, rats with retrosplenial cortex lesions performed an attentional set-shifting task, previously shown to be sensitive to lesions centred in prelimbic cortex [28]. These medial frontal lesions produce a selective deficit on extradimensional set-shifting, the ability to switch from one class of reinforced cues to another [28], while more selective anterior cingulate lesions can impair intradimensional set-shifting [29]. The latter experiment also examined the retrosplenial (posterior cingulate) cortex, finding that restricted lesions centred on mid anterior-posterior levels within the area can also retard intradimensional shifts [29]. In Experiment 2, a second cohort of rats with retrosplenial lesions was tested on a strategy switch task in an automated chamber. This task involved learning and then shifting between visual-based and response-based discriminations, switches that are sensitive to inactivation of the medial frontal cortex in rats (Ragozzino et al. [31]; Floresco et al. [30]). Both Experiments 1 and 2 included a reversal stage as this form of behavioural change can be dissociated from shifting attention from one domain to another [28], [32].

Lesions in the anterior cingulate cortex impair effort-based decision making, whilst other medial frontal cortex lesions can spare such tasks [33]. We, therefore, trained the cohort of rats from Experiment 2 on an operant cost-benefit discrimination task (Experiment 3). Such tasks pit reward size against the effort required to gain that reward [34]. Finally, a third cohort of rats with retrosplenial lesions was trained on reinforced matching-to-place in a T-maze (Experiment 4). Rats with prelimbic cortex lesions show impaired acquisition as they persist with the dominant preference to nonmatch and then spend longer resorting to a side preference, before learning to match [35], [36]. Anterior cingulate lesions also extend the period of nonmatching, but the animals then switch at a rapid rate once that innate tendency has extinguished [35]. Taken together, the various behavioural tasks were selected to determine whether retrosplenial cortex lesions have effects similar to those seen after lesions in the anterior cingulate or prelimbic cortices.

## 2 Experiment 1: attentional set–shifting

The first experiment consisted of a series of discriminations that were completed in a single session [37], [38]. The initial discriminations helped to establish within-dimensional attention (intradimensional shifts), while subsequent discriminations involved an extradimensional shift, followed by a reversal of the preceding discrimination.

### 2.1 Methods

#### 2.1.1 Subjects

Experiment 1 involved a single cohort (Cohort 1) of 28 male Lister hooded rats (Harlan, Bicester, UK) weighing between 278 and 387 g at the time of surgery. Animals were housed in pairs under diurnal light conditions (14 h light/10 h dark). Behavioural testing occurred during the light phase. The rats were handled daily for a week prior to surgery and then randomly assigned to one of two surgical groups: retrosplenial cortex lesions (RSC1, n = 16) or surgical shams (Sham1, n = 12). All procedures were in accordance with the UK Animals (Scientific Procedures) Act, 1986 and EU directive (2010/63/EU), as well as being approved by local ethical committees at Cardiff University. Prior to Experiment 1, the rats had been tested on a spatial discrimination task and a spontaneous object recognition task [39]. The current experiment began eight months after surgery.

#### 2.1.2 Surgical procedures

Prior to surgery, rats received a subcutaneous injection of 0.06 ml Metacam (Boehringer Ingelheim, Alkmaar, NL, USA) and an intraperitoneal (i.p.) injection of 0.1 ml Millophylline (Arnolds Veterinary Products Ltd, Shrewsbury, UK). They were then deeply anaesthetised with an i.p. injection of sodium pentobarbital (60 mg/kg pentobarbital sodium salt; Sigma-Aldrich, U.K.). The rat was placed in a stereotaxic frame with the nose bar at +5.0 (David Kopf Instruments, Tujunga, CA, USA). The scalp was retracted and the cortex along the midline exposed by a bilateral craniotomy, extending from bregma to lambda.

Lesions were produced by injecting 0.09 M *N*-methyl-d-aspartate solution (NMDA; Sigma, Poole, UK), dissolved in phosphate buffer (pH 7.2), into seven bilateral injection sites via a 1 μl Hamilton syringe at a rate of 0.05 μl per minute (Bonaduz, Switzerland). The injection coordinates were measured from bregma along the anterior-posterior (AP) axis, from the central sinus along the lateral-medial (LM) axis, and from the surface of the cortex for the dorsal-ventral (DV) axis. The stereotaxic coordinates at each of the seven sites were as follows: AP −1.6, LM ± 0.4, DV-1.3; AP-2.8, LM ± 0.5, DV-1.3; AP-4.0, LM ± 0.5, DV-1.3; AP-5.3, LM ± 0.5, DV-2.6; AP-5.3, LM ± 0.9, DV-1.6; AP-6.6, LM ± 1.0, DV-2.0; AP-7.5, LM ± 1.1, DV-1.3. The three most rostral coordinates received injections of 0.25 μl NMDA, the next three pairs of sites received 0.26 μl, with 0.1 μl NMDA in the most caudal site.

#### 2.1.3 Apparatus

**Fig. 1:**
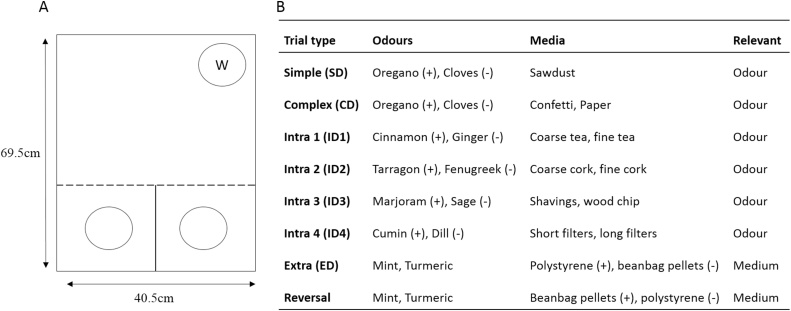
Set-shifting task (Experiment 1). (A) Schematic of the arena used for the set-shifting experiment. Approximately one third of the length of the box was divided into two smaller compartments. The remaining area of the box was a single open space. The two smaller sections could be separated from the larger area by a removable black Perspex panel. A glass pot (depicted by the clear circles) containing the digging medium was placed in each of the two smaller areas. A third identical glass pot containing water was placed against the opposite wall of the larger area (depicted by the “W”). (B) Examples of possible stimulus pairings for each stage of the attentional set-shifting task. A rewarded stimulus is indicated with + while − indicates the nonrewarded stimulus in each pair. Abbreviations: extra, extradimensional shift; intra, intradimensional shift.

Rats were tested in an opaque, black Perspex box (69.5 cm long x 40.5 cm wide x 18.6 cm deep). The box was divided into two smaller compartments along approximately one third of its length, with the remaining area creating a single open space (Fig. 1A). Removable opaque Perspex panels controlled access to the two smaller sections from the larger area. Each of the three sections was covered by a separate, hinged transparent Perspex lid. A glass pot (75 mm in diameter, 45 mm deep), containing the digging media, was placed in each of the two smaller areas. Given the duration of the test session, a third identical pot, containing water, was placed against the wall of the larger area, furthest from the digging chambers (see Fig. 1A). Rats were carried to and from the testing room in individual, opaque boxes.

#### 2.1.4 Behavioural training

##### 2.1.4.1 Pre-training

Three days prior to testing, each rat was habituated to the full test arena for 10 min (no glass pots were present). On the second pre-training day, rats were returned to the arena for another 10 min. Now, both the smaller chambers were closed off by the removable panels and the three glass pots were in place. The animal was initially placed in the larger chamber and the dividing panels were raised and lowered at intervals (approximately every 2 min). During this session both of the pots in the smaller chambers (i.e., the digging pots) were filled with clean bedding sawdust and baited with half a Cheerio (Nestle, UK) and rats were trained to retrieve the food from the pots. Note that this stage was acquired relatively quickly because all the animals had been previously trained on a similar task [12]. The day before testing, rats were again placed in the arena and pre-exposed to each of the test stimuli in turn. Odours were mixed with bedding sawdust and all the different digging media were presented without odours added. For all stimulus presentations, rats were required to retrieve a buried Cheerio from each pot.

##### 2.1.4.2 Test

On the Test Day, rats received a series of two-choice (forced) discriminations in which only one stimulus dimension (i.e., odour or texture; counterbalanced across rats) signalled reinforcement (see Fig. 1B for examples). Each rat completed all discriminations in a single session. For each discrimination, the two digging pots contained different media and/or odours and a single cheerio was placed in one of the two pots. The location of the reinforced pot (i.e., in the left or right chamber) was pseudo-randomly allocated across trials.

At the start of each trial, a rat was placed in the large compartment with the dividing doors restricting access to the both of the smaller chambers. A trial started once these doors were removed and the rat had 10 min to find the reward. A ‘choice’ was defined as the rat breaking the surface of the digging medium with its paws or nose. If the rat chose the non-reinforced pot, the trial was marked as incorrect. For the first four trials of each stage, if a rat made an incorrect choice, it was allowed access to the correct pot to uncover the reward. On subsequent incorrect trials (i.e., digging in a nonbaited pot), the trial was terminated once the rat returned to the large waiting area of the arena. The pots were re-baited during a 5 s ITI period. A rat did not move onto the next discrimination stage until it had reached a criterion of six correct choices in a row. Trials were scored by an experimenter blind to the group identify of the rat.

For the initial, simple discrimination (SD), the digging pots were filled with either sawdust infused with two different scents or with two different digging media (without odour). Only one odour (or one medium) was reinforced. In the subsequent, complex discrimination (CD) stage, the same odour or texture as for the simple discrimination trials was reinforced, but now the irrelevant dimension, texture or odour respectively, was also added (see Fig. 1B).

The next four stages consisted of intradimensional training (ID1, ID2, ID3, and ID4) in which different compound stimuli were presented for each discrimination, with the relevant dimension (i.e., odour or texture) remaining constant. Thus, for the first six stages of the task (SD, CD, ID1, ID2, ID3, and ID4) the rats were required to attend to only one stimulus dimension, while ignoring the other non-reinforced dimension (see Fig. 1B). This training was designed to encourage the formation of an attentional set (i.e., always attend to one dimension and ignore the other, irrelevant dimension).

After the fourth intradimensional discrimination (ID4), an extradimensional (ED) shift was introduced. Again, different compound stimuli were presented, however, now the previously irrelevant dimension was reinforced (see Fig. 1B). Consequently, rats had to attend to a different dimension in order to solve the discrimination. Finally, the reward contingencies established during the ED task were reversed (REV) such that the previously non-reinforced stimulus was now reinforced and *vice versa* (see Fig. 1B).

#### 2.1.5 Histology

After behavioural testing, rats were deeply anaesthetized with sodium pentobarbital (60 mg/kg, i.p.; Euthatal; Merial Animal Health, Harlow, UK) and transcardially perfused with 0.1 M phosphate-buffered saline (PBS) followed by 4% paraformaldehyde in 0.1 M PBS (PFA). The brains were removed and placed in PFA for 4 h before being transferred to 25% sucrose overnight at room temperature, with gentle agitation. A one-in-four series of coronal sections (40 μm) was cut on a freezing microtome and stained using the Nissl stain, cresyl violet.

Descriptions of the rat retrosplenial cortex follow those of van Groen and Wyss [3], [40], [41]. In this nomenclature, the dysgranular cortex (area 30) is designated Rdg, while the granular cortex (area 29) is subdivided into a more dorsal area, Rgb and a more ventral, caudal area, Rga [3].

#### 2.1.6 Data analysis

Trials to criterion were analysed using mixed ANOVAs. Discrimination “Stage” (eight levels) provided a repeated-measures factor, while “Group” was the between-subjects factor. Switch cost values were calculated for the extradimensional shift by subtracting the trials to criterion for the final intradimensional discrimination (ID4) from the trials to criterion for the ED shift stage.

Data from all of the four experiments described here were analysed in R (V 3.3.2). All ANOVAs were conducted in the ‘afex’ package using the ‘aov_ez()’(Type 3 ANOVA) function (Singmann et al. [42]). Where violations of sphericity occurred, degrees of freedom were adjusted using the Greenhouse-Geisser correction.

### 2.2 Results

#### 2.2.1 Histology

**Fig. 2:**
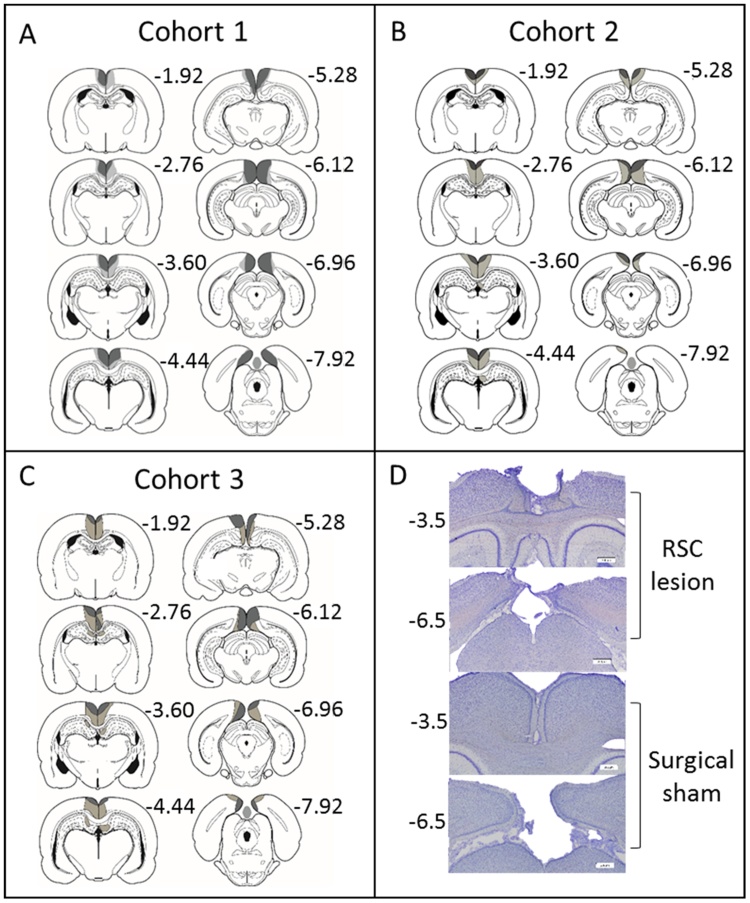
Histology. (A–C) Location and extent of the retrosplenial cortex (RSC) lesions for each of the three cohorts. The cases with the largest (pale grey) and smallest (dark grey) cortical lesions are depicted on a series of coronal sections The numbers refer to the approximate distance, in mm, of each section caudal to bregma [67]. (D) Photomicrographs from a representative lesion (top two rows) and a surgical sham case (bottom two rows) from groups RSC2 and Sham2. Scale bars represent 200 μm.

Of the 16 rats in group RSC1, three were excluded due either to excessive sparing of retrosplenial cortex or because of marked bilateral damage to the hippocampus. In the remaining 13 RSC1 rats, the lesions were largely as intended as extensive cell loss and gliosis was seen throughout the retrosplenial cortex in both the granular and dysgranular subregions (Fig. 2A). Three animals had restricted damage or gliosis in the most dorsal medial tip of the CA1 subfield of the hippocampus (two unilateral) below the rostral retrosplenial cortex. In the remaining case, the bilateral CA1 damage was extremely restricted. Of these three cases, the maximum extent of anterior–posterior hippocampal damage was limited to 600 μm. In addition, seven animals, including the three with CA1 damage, had slight unilateral thinning of the medial blade of the dentate gyrus just caudal to the splenium. Nine animals had partial sparing of Rga, particularly at its caudal limit. Four rats also had some limited sparing of Rgb. One rat had slight damage to the anterior cingulate cortex at the junction with retrosplenial cortex, and two showed limited unilateral damage to the secondary motor cortex, lateral to the retrosplenial cortex. A restricted area of gliosis was observed at the junction of the anterior medial and anterior ventral nuclei, as is consistently observed after extensive retrosplenial lesions [9], [43], [44]. Following histological analyses the final group sizes were; RSC1 n = 13, Sham1, n = 12.

#### 2.2.2 Behaviour

**Fig. 3:**
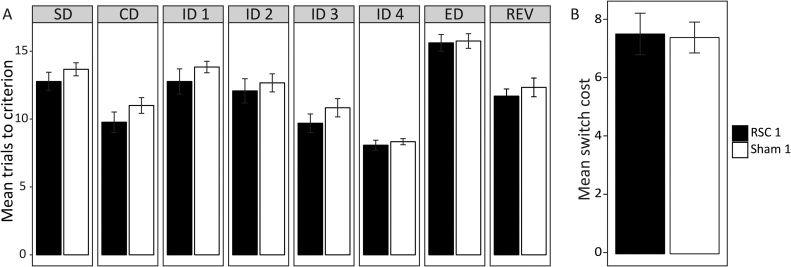
Attentional set-shifting (Experiment 1). (A) Mean trials to criterion across the eight discrimination stages. (B) Mean extradimensional switch cost values for the two groups. Switch cost values were calculated by subtracting the trials to criterion for the final intradimensional discrimination (ID4) from the trials to criterion for the extradimensional (ED) shift stage. Error bars show ±SEM. Other abbreviations: CD, complex discrimination; REV, reversal; SD, simple discrimination (see Fig. 1).

When all eight discrimination stages were analysed together, there was no overall group difference for trials to criterion (*F*_1,23_ = 1.74 *p* = 0.20; see Fig. 3A and Supplementary Table 1). Likewise, there was no interaction between Stage and Group (*F* < 1), though there was a main effect of Stage (*F*_7161_ = 36.93 *p* < 0.001).

Further ANOVAs, conducted on the intradimensional shift stages (ID1-4), confirmed the improvement in performance across successive discriminations (*F*_3,69_ = 38.48 *p* < 0.001; Fig. 3A). This improvement in discrimination performance is consistent with enhanced intradimensional attention, i.e., successful set formation. At the same time, there were no Group differences in the number of trials to criterion (*F*_1,23_ = 1.24 *p* = 0.28). Moreover, there was no interaction between Group and ID stage (*F *< 1), indicating that the acquisition of the intradimensional attentional set was equivalent in the two groups.

To examine more closely the effect of the extradimensional shift (ED), the switch-cost difference (i.e., ED stage − ID4 stage) was calculated for each animal (Fig. 3B). There was no group difference on this measure (*t *< 1). Furthermore, both the RSC1 (*t_12_* *=* 10.58 *p <* 0.001) and Sham1 (*t_11_* = 14.03 *p* < 0.001) groups showed a significant switch cost (i.e., increased trials to criterion for the ED stage relative to the ID4 stage). Moreover, there was no lesion effect on the subsequent reversal stage (*t* < 1, Fig. 3A). Note, comparable analyses based on errors rather than trials to criterion gave the same pattern of results throughout.

## 3 Experiment 2: strategy-shift

The first experiment found no evidence that retrosplenial cortex lesions disrupt the acquisition of an intradimensional learning set, or affect the ability to switch dimensions. To examine these forms of learning more fully, the next experiment used an automated chamber to test the ability to shift between response-based and visual-based discriminations. The task was closely based on one described by Floresco [30].

### 3.1 Methods

#### 3.1.1 Subjects

Experiment 2 involved a second cohort (Cohort 2) of 30 male Lister Hooded rats (ENVIGO, Bicester, UK). At the time of surgery, the rats weighed 309–356 g. Animals were housed in groups of four under diurnal light conditions (14 h light/10 h dark), with all behavioural testing during the light phase. Prior to surgery, the rats were handled daily for a week and then randomly assigned to one of two surgical groups: retrosplenial cortex lesions (RSC2, n = 15) or surgical shams (Sham2, n = 15).

#### 3.1.2 Surgical procedures

The general surgical procedures followed Experiment 1 but only six bilateral injections of NMDA were made. This refinement was made in order to reduce the surgery time and to minimise the risk of excessive bleeding. Rats were deeply anaesthetised (1 ml/kg, i.p. injection) with 6% sodium pentobarbital solution (Ceva Animal Health, Libourne, France). Anaesthesia was then maintained with isoflurane (∼0.5%) in O_2_ for the duration of the surgery. Injection coordinates were calculated in the same way as Experiment 1 except that the dorsal-ventral (DV) coordinates (in mm) were taken from the height of the dura. The stereotaxic coordinates at each of the six sites were as follows: (#1) −1.8 (AP), ±0.5 (ML), −1.0 (DV); (#2) −2.8 (AP), ±0.5 (ML), −1.1 (DV); (#3) −4.0 (AP), ±0.5 (ML), −1 (DV); (#4) −5.3 (AP), ±0.5 (ML), −2.5 (DV); (#5) −5.3 (AP), ±0.9 (ML), −1.4 (DV); (#6) −6.6 (AP), ±0.9 (ML), −1.8 (DV). A volume of 0.25 μl of NDMA was injected at the first three pairs of sites (#1-3) while 0.27 μl was injected in the remaining sites. All animals also received an injection of atropine (0.06 ml of a 600 μg/ml solution, Martindale Pharma, Brentwood, UK).

#### 3.1.3 Apparatus

All instrumental training was conducted in a set of eight operant boxes (Med Associates Inc., St Albans, VT), each measuring 240 mm high x 240 mm deep x 300 mm wide. The boxes were arranged in two rows of four. Each box had two aluminium walls, with a clear Perspex front, back, and ceiling. The grid floor comprised 19 parallel stainless steel bars spaced 16 mm apart. Each operant box was housed in its own sound and light attenuating chamber.

During training, sucrose pellet reinforcers (45 mg; P. J. Noyes, Lancaster, NH) were delivered into a recessed food magazine situated in the centre of the right-hand wall of the operant box. The magazines were fitted with a pair of infra-red detectors that recorded magazine entry and exit. Retractable flat-panel levers were inserted to the left and/or right of the magazine at the start of each session and retracted when the session ended. Equipment control and data recording were via an IBM-compatible PC equipped with MED-PC software (Med Associates Inc., St Albans, VT).

#### 3.1.4 Behavioural training

##### 3.1.4.1 Pre-training

Before being introduced to the operant chambers, rats were habituated to the sucrose pellets in their home cages. The following day, all animals received a single session of magazine training during which reinforcers were delivered into the food magazine on a variable interval 60 s schedule (i.e., on average, one pellet per minute). After magazine training, the animals completed two sessions of continuous reinforcement (one on each lever, counterbalanced across animals), during which one lever was inserted into the operant chamber and every lever press was reinforced. The animal was required to press the lever at least 50 times in 30 min before proceeding to the next stage. If this criterion was not met, the animal completed additional sessions on that lever until they reached criterion.

In the final stage of pre-training, either the left or the right lever was presented on a given trial. The side on which the lever was presented was random for the first trial of a pair and the opposite lever was then presented on the subsequent trial. Trials commenced with illumination of the house light and the insertion of the lever. If the animal made a response within 10 s of the lever being inserted, a pellet was delivered, the lever retracted and, after 4 s, the house light was switched off. If the animal failed to make a response within 10 s, the lever was retracted, the house light switched off and the trial counted as an omission. Each session consisted of 90 trials (45 left lever/45 right lever) and all animals completed at least four sessions. If an animal made more than five omissions in the final session it received another session until this criterion was met (i.e., ≤5 omissions).

##### 3.1.4.2 Discrimination training

**Fig. 4:**
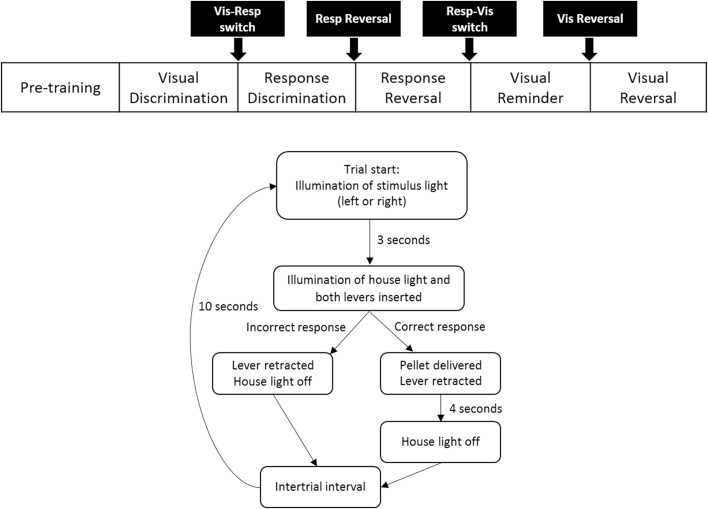
Strategy-shift task (Experiment 2). Schematic showing the order of the discrimination stages and basic trial structure for the strategy-shift task. The black boxes show the points at which a new discrimination strategy was introduced.

All animals learnt two discrimination strategies (visual and response discrimination stages), which required the use of different cues to earn food reinforcement. In addition, animals completed sessions in which each of these strategies was reversed (visual reversal and response reversal stages). The order in which the discrimination stages were completed was the same for all animals (Fig. 4).

Each session terminated after the animal had completed at least 60 trials and had reached a performance criterion of 10 consecutive correct responses, or after 120 trials. All animals received at least two sessions on each discrimination stage and needed to be at criterion over two sessions, with the additional stipulation that they made fewer than 20% errors on the final session, before progressing to the next stage. This requirement helped to ensure that the animals had sufficiently learnt the discrimination in order for that session to be used as a baseline from which to calculate the switch-cost ratio.

##### 3.1.4.3 Visual-Guided discrimination

All animals completed the Visual-Guided Discrimination first (Fig. 4). Here, one of the stimulus lights (left or right) was illuminated at the start of each trial. Three seconds later both levers were inserted into the operant chamber and the house light illuminated. A response on the lever below the illuminated stimulus light (correct response) resulted in the delivery of a single pellet, the extinguishing of the stimulus light, and the retraction of the levers. After 4 s the house light was extinguished, signalling the start of the 20 s inter-trial interval (ITI). Following an incorrect response (i.e., a response on the other lever) the chamber immediately reverted to the ITI state. If the animals failed to make a response within 10 s of the trial starting, the chamber reverted to the inter-trial state (Fig. 4). The order of the correct lever (left or right) was random for the first trial of a pair, while in each pair of trials both the left and right lever were presented. For example, on the first trial of a session there was an equal probability of either the left or right lever being presented, whilst on the second trial the opposite lever was always presented. Then, on the third trial, the probability of the either lever being presented was again equal, and so on for the entire session.

##### 3.1.4.4 Response discrimination

The Response Discrimination trials were essentially the same as those in the Visual-Guided Discrimination stage (Fig. 4). However, now, either the left or right lever was designated as the ‘correct’ lever (counterbalanced across animals); only responses on this lever were reinforced. The stimulus light was still presented above one of the levers so that, over the course of the session, for half of the trials the light was illuminated above the correct lever (‘Congruent trials’) and for remaining trials the light was above the incorrect lever (‘Incongruent trials’).

##### 3.1.4.5 Response reversal training

After reaching criterion on the Response Discrimination, the levers were reversed such that the previously incorrect lever was now reinforced and *vice versa*. All other conditions remained the same as in the previous stages.

##### 3.1.4.6 Visual reversal training

After reaching criterion on the Response Reversal, training was paused to allow all animals to complete this stage. Thus, to ensure that all animals were at a similar stage of performance, before progressing on to the next stage, they all completed two Response Reversal reminder sessions during which the same lever was reinforced as for the Response Reversal stage. If they reached criterion on both sessions they moved on to the next stage.

The training sessions now reverted back to the initial Visual-Guided Discrimination strategy. Once a rat performed at criterion, the strategy reversed such that now the animal had to learn that the stimulus light signalled the incorrect lever.

#### 3.1.5 Data analysis

Mean trials and errors to criterion for each stage were compared between the groups using a mixed ANOVA with the repeated (within) factor Stage and the between-factor Group. To examine more closely the effects of a strategy switch on the groups, the first session of each stage (i.e., when a new strategy was introduced) was split into blocks of 10 trials and the first six blocks (i.e., 60 trials) were analysed separately for each stage using a mixed ANOVA with the factors Block (1–6) and Group.

Switch cost values were calculated for each animal by subtracting the total number of errors during the first session of a given stage from the total errors during the final session of the previous stage. For each group, at each stage, these switch cost differences were analysed using one sample *t*-tests (mean switch cost > 0).

In addition, for the Response Discrimination and Visual Reminder stages (i.e., stages where a strategy shift was required, as opposed to a simple reversal), trials were classified according to whether or not the correct lever was the same as it would have been for the previous discrimination (i.e., Congruent trials) or different (i.e., Incongruent trials). For example, during the Response Discrimination stage the light was illuminated above the correct lever (i.e., a Congruent trial) on half of the trials, meaning that the discrimination could be solved using the previously learnt strategy (i.e., press the lever with the light above it). Conversely, in order to select the correct lever on Incongruent trials (i.e., when the light was illuminated above the incorrect lever) the animal had to inhibit the previously learnt strategy. Therefore, errors made on Incongruent trials were categorised as ‘Perseverative’. Perseverative errors rates were analysed as a fraction of overall errors for the first six blocks (i.e., 60 trials) of the first Response Discrimination session and, in a separate ANOVA, for the first six blocks of the first Visual Reminder session.

### 3.2 Results

#### 3.2.1 Histology

Two animals from group RSC2 were excluded due to substantial bilateral damage in dorsal CA1. Of the remaining 13 animals, five had lesions centred in the dysgranular retrosplenial cortex. One of these five cases had almost complete sparing of the granular cortex both anterior and posterior to the splenium. In the remaining four cases, some cell loss was evident in the deeper layers of the granular cortex. A sixth animal had appreciable unilateral sparing in granular retrosplenial cortex anterior to the splenium (Fig. 2B).

The remaining seven RSC2 animals showed considerable bilateral cell loss in both the granular and dysgranular cortices, both anterior and posterior to the splenium (Fig. 2B). In some cases, this cell loss was particularly evident in the more superficial cell layers (Fig. 2). In two of these cases there was a small amount of sparing in granular retrosplenial cortex close to the anterior cingulate border. Caudal to the splenium, there was often some sparing in the most caudal parts of area Rga. Three cases had extremely restricted, unilateral cell loss in the dorsal medial CA1, below the rostral retrosplenial cortex. In one case, the lesion encroached into the most caudal anterior cingulate cortex. Following histological analyses the final group sizes were; RSC2 n = 13, Sham2, n = 15.

#### 3.2.2 Behaviour

In addition to histological exclusions, a further four animals (two Sham2 and two RSC2) were excluded due to technical problems affecting the first Visual-Guided Discrimination (the lights in one of the operant chambers were found to be incorrectly configured). In total, data from 11 RSC2 and 13 Sham2 animals were compared. Of the 11 RSC2 animals, four had bilateral sparing in granular retrosplenial cortex (as described above). Therefore, to assess the effect of granular sparing, the RSC2 group was split by lesion size (Complete: n = 7; Dysgranular: n = 4). There were no differences between the groups in the number of errors or trials to criterion (*F <* 1). Furthermore, there was no interaction with Stage for either errors (*F*_1.7,15.4_ = 1.08 *p* = 0.35) or trials to criterion (*F <* 1). Therefore, for all further analyses combined these two subgroups.

#### 3.2.3 Overall comparisons

When all five discrimination stages were analysed together, there was no overall difference in either overall errors or trials to criterion between the groups. Furthermore, there was no interaction between Group and Stage for either of these measures (all *F <* 1).

#### 3.2.4 Within-stage comparisons

All rats completed a minimum of 60 trials on the first session of each discrimination stage (analysed as six blocks of ten trials). Therefore, to examine more closely the effects of a strategy switch, these 60 trials were split into blocks of 10 trials and error rates over these blocks were analysed separately for each stage, using a mixed ANOVA with the factors Block (1–6) and Group.

**Fig. 5:**
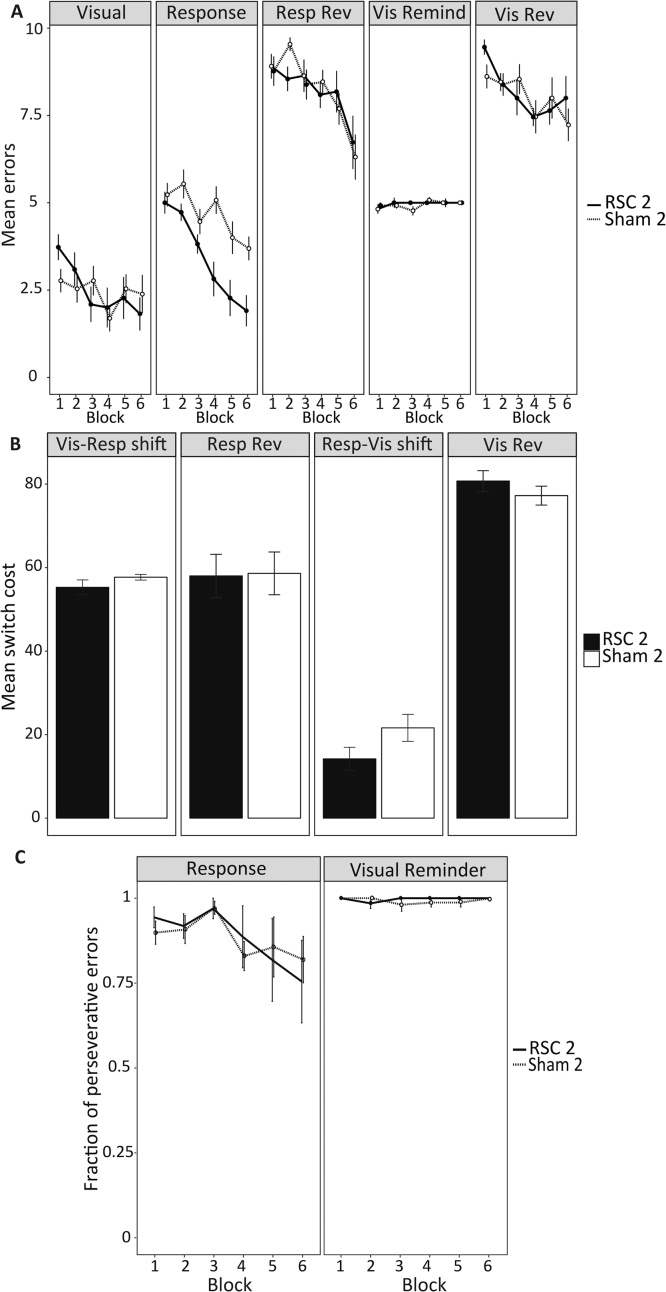
Strategy-shift (Experiment 2). (A) Mean errors made during the first 60 trials (6 block of 10 trials) of the first session of each discrimination stage. (B) Mean switch cost values for each stage. Switch cost values were calculated for each animal by subtracting the total errors during the first session of a given stage from the total errors during the final session of the previous stage. (C) Fraction of perseverative errors. Trials were classified according to whether or not the correct lever was the same as it would have been for the previous discrimination (i.e., Congruent trials) or different (i.e., Incongruent trials). Errors made on incongruent trials were categorised as ‘Perseverative’. Perseverative errors are presented as a fraction of overall errors for the first six blocks of the first Response Discrimination session (left panel) and the first session of the first Visual Reminder session (right panel). Error bars show ±SEM. Abbreviations: Rev, reversal.

Error rates decreased across these first six blocks of the Visual-Guided Discrimination (*F*_5110_ = 4.16 *p* = 0.002), with no difference in error rates between the groups across the same blocks (*F <* 1) and no interaction between Group and Block (*F*_5110_ = 1.78 *p* = 0.12; Fig. 5A). For the first session of the Response Discrimination there was a main effect of Group (*F*_5,22_ = 18.45 *p <* 0.001), with the RSC2 group making fewer errors than the Sham2 group (Fig. 5A). There was also an interaction between Group and Block (*F*_5110_ = 2.48 *p* = 0.04). Simple effects analysis revealed that RSC2 animals made significantly fewer errors than Sham animals in blocks four (*F*_1,22_ = 13.77 *p* = 0.001), five (*F*_1,22_ = 6.50 *p* = 0.02) and six (*F*_1,22_ = 11.06 *p* = 0.003; see Fig. 5A), consistent with a faster rate of acquisition. Note that because all rats were trained to a criterion, performance was matched prior to the next stage (Response Reversal).

In the first session of the Response Reversal stage, error rates decreased across the six blocks (*F*_3.4,74.7_ = 11.87, *p <* 0.001), with no effect of Group or Group by Block interaction (F < 1). Likewise, overall error rates decreased on the Visual Reversal stage (*F*_5110_ = 5.04 *p* = 0.04), but not for the Visual Reminder stage (*F*_5110_ = 1.30 *p* = 0.28). There was no main effect of Group, or Group by Block interaction, on error rates for either of these discrimination stages (all *p >* 0.05; Fig. 5A).

#### 3.2.5 Switch cost comparisons

Importantly, both groups showed a significant switch cost (total errors during the final session of a given stage − total number of errors during the first session of the subsequent stage) at all discrimination stages (all *p* ≤ 0.001, one sample *t* test; Fig. 5B). There was no main effect of Group on the switch cost differences and no interaction between Stage and Group (*F*_1.8,39.2_ = 1.14 *p* = 0.33*;* Fig. 5B).

#### 3.2.6 Congruent and incongruent trials: error analysis

Congruent trials were those in which the correct response was accompanied by a light above the same lever (i.e., congruent with the previous discrimination). Errors made on incongruent trials were therefore termed “perseverative”. Whilst the overall number of errors made during the first 60 trials of the first Response discrimination stage differed between the groups, there was no overall difference between the groups in the fraction of preservative errors made during this period (*F <* 1) nor was there a Group by Block interaction (*F <* 1; Fig. 5C). Similarly, there was no group difference in the fraction of perseverative errors made during the Visual Reminder stage or Group by Block interaction (*F <* 1; Fig. 5C).

## 4 Experiment 3: cost-benefit discrimination

**Fig. 6:**
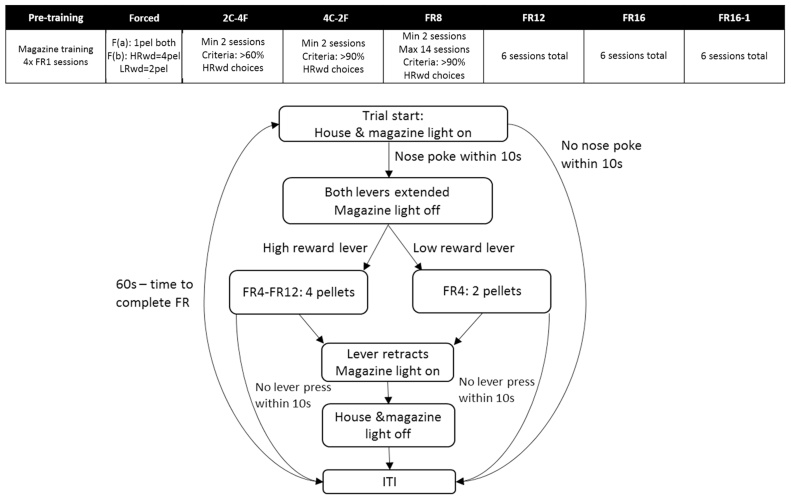
Cost-benefit task (Experiment 3). Schematic showing the order of all stages, and basic trial structure, of the cost-benefit task. Abbreviations: C = choice trials; F = forced trials; FR = fixed ratio.

Experiment 2 revealed a selective lesion effect, with the RSC2 animals making fewer errors after switching from a visual-cue to a response-based strategy. Lesions in the anterior cingulate cortex impair effort-based decision making, whilst medial prefrontal cortex lesions can spare such tasks [33]. We, therefore, trained all rats in the RSC2 cohort on an operant task based on similar principles (Fig. 6).

### 4.1 Methods

#### 4.1.1 Subjects

For subject information and details of surgery see Experiment 2.

#### 4.1.2 Apparatus

The same set of operant chambers were used as for Experiment 2. For this experiment, food pellets were used for reinforcement, rather than sucrose, to reduce the transfer of learning from the strategy-shift task.

#### 4.1.3 Behavioural training

##### 4.1.3.1 Pre-training

At the start of training, all animals completed a single 30 min magazine training session, during which food pellets were delivered on a variable interval 60 s schedule. The magazine light was illuminated for 6 s following pellet delivery. During the next session, rats were trained to press the levers on a fixed ratio 1 schedule (FR1, one lever press = 1 pellet). The house light remained on for the entire session and the magazine light was illuminated as a pellet was delivered, the magazine light remaining lit for 4 s. Each animal received a total of four 15 min sessions of FR1 training, two sessions on each lever, on alternate days (counterbalanced across subjects).

##### 4.1.3.2 Forced sessions

The start of a trial was signalled by illumination of the house light and the magazine light (Fig. 6). A time limit of 10 s for latency to the first magazine entry and for all lever press responses was also introduced: if an animal failed to make a magazine response within 10 s of the trial start, or if they failed to make a lever press within 10 s after the initial magazine entry or previous lever response, the trial was terminated and counted as an omission. There was a 60 s time out after an omission trial during which all lights were extinguished and the levers retracted. After the first magazine entry response, the magazine light was extinguished and either the left or right lever was extended. A single lever press resulted in the delivery of a food pellet and the illumination of the magazine light. The magazine light remained on for six seconds after which the trial was ended and all lights extinguished. After an ITI of four seconds, the house light and magazine light were illuminated, signalling the start of a new trial. Animals completed 48 trials, with the left lever presented on half the trials and the right lever presented on the other half. For the first trial of a pair there was an equal probability of either the left or the right lever being presented, with the opposite lever presented on the second trial of a pair. The subsequent three sessions followed the same basic structure but the ITI was increased from four seconds to 10 s–20 s, respectively. Similarly, the FR was increased from one to two to four lever presses.

##### 4.1.3.3 Cost-Benefit discrimination: forced sessions

For all subsequent sessions (Fig. 6), either the left or right lever was designated as the high reward lever (HRwd) and the other lever as low reward (LRwd). The position of the HRwd and LRwd (i.e., left or right) was counterbalanced between subjects and remained the same for the duration of the experiment. For the first four sessions of this phase of training, all trials were forced (i.e., either the left or the right lever was presented) and the trial structure was the same as for the final session of the previous stage. Initially both the LRwd and HRwd levers were reinforced on an FR4 schedule but, for trials in which the HRwd lever was presented, the animals received 4 food pellets whilst only two pellets were delivered for LRwd trials.

A time limit of 10 s for latency to the first magazine entry and for all lever press responses was also introduced: if an animal failed to make a magazine response within 10 s of the trial start, or if they failed to make a lever press within 10 s after the initial magazine entry or previous lever response, the trial was terminated and counted as an omission. There was a 60 s time out after an omission trial during which all lights were extinguished and levers retracted. The standard ITI remained at 20 s for the first session of this stage and was increased to 30 s and then 45 s for the second and third sessions, respectively. In the final session of this stage the ITI was 60 s minus the time taken for the FR4 schedule to be completed (Fig. 6).

##### 4.1.3.4 Cost-benefit discrimination: choice sessions

All subsequent sessions consisted of a mix of forced and choice trials (Fig. 6). Initially, there were two choice trials for every four forced trials. On choice trials, both levers were presented at the start of the trial and, after the first lever press, the other lever was immediately retracted. All animals completed at least two sessions of this stage and were given an additional session if they failed to make >60% HRwd choices on the second session. They then moved on to the next stage, in which they were given four choice trials for every two forced trials. To start with, the fixed ratio was set at FR4 for both levers. Animals completed at least two such sessions and once they made an average of > 90% HRwd choices across two sessions, an effort differential was introduced for the HRwd vs LRwd lever.

Initially, the fixed-ratio for the low reward (LRwd) lever was kept at FR4, whilst the fixed-ratio for the high reward (HRwd) lever was increased to FR8. Again, the criterion for completing this stage was set at an average of >90% HRwd choices across two sessions. If an animal failed to reach this criterion after 14 FR8 sessions, their data were excluded from the analysis.

Once the criterion had been reached, the HRwd fixed ratio was increased to FR12. All animals that made it to this stage completed six sessions, after which the HRwd fixed-ratio was increased to FR16. The animals completed a further six sessions of this stage before moving on to the final stage, during which the reinforcement differential between the two levers was increased by reducing the LRwd lever reinforcement rate from two pellets to one (FR16-1; see Fig. 6).

#### 4.1.4 Data analysis

For each session, the fraction of HRwd choices was calculated by dividing the number of HRwd choices by the total number of choice trials (i.e., 32). These values were then compared between the groups for each stage separately. For the FR8 stage, because the animals completed different numbers of sessions, HRwd choice fractions were averaged across session and compared between the two groups using an independent samples *t*-test. For the FR12, FR16, and FR16-1 stage, HRwd choice fractions across sessions were analysed between groups using a mixed ANOVA.

### 4.2 Results

See Experiment 2 and Fig. 2B for details of lesion placement and exclusions. A further three animals were excluded from the analysis (one RSC2 and two Sham2) because they failed to reach the FR8 stage criterion. Final group sizes for Experiment 3 were; RSC2 n = 12; Sham2 n = 13. Of the 12 RSC2 animals, four had bilateral sparing in granular retrosplenial cortex (as described above). Therefore, to assess the effect of granular sparing, the RSC2 group was split by lesion size (Complete: n = 8; Dysgranular: n = 4). There were no differences between these two groups, or interactions with session, in the mean HRwd choice fractions for the any of the stages (all *p <* 0.05).

#### 4.2.1 Behaviour

**Fig. 7:**
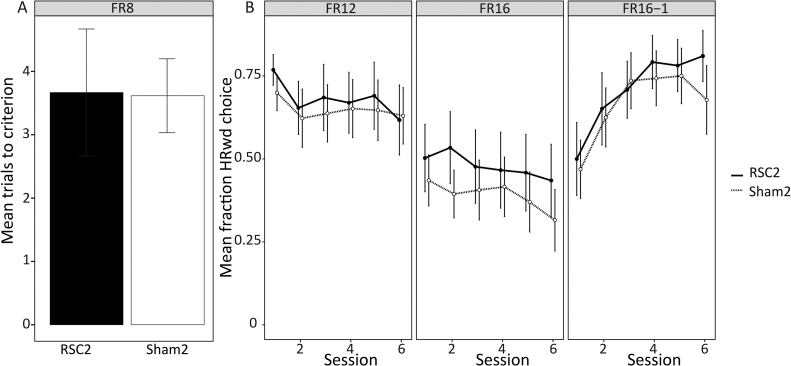
Cost-benefit task (Experiment 3). (A) Mean trials to criterion for the FR8 stage. Criterion = average of >90% HRwd choices across two sessions. (B) Mean fraction of high reward (HRwd) lever choices across the six sessions for the FR12, FR16 and FR16-1 stages. The fraction of HRwd choices was calculated by dividing the number of HRwd choices by the total number of choice trials. Error bars show ±SEM.

The mean fraction of HRwd choices made during the first session of each of the stages was analysed using a one-sample *t*-test to determine if it was above the chance score of 0.5. Both groups were significantly above chance for the first FR8 session (RSC3: *t_11_* *=* 7.44 *p <* 0.001; Sham3: *t_12_* *=* 14.27, *p <* 0.001; Fig. 7A) and the first FR12 session (RSC3: *t_11_* = 5.8, *p <* 0.001; Sham3: *t_12_* *=* 3.67, *p* = 0.001; Fig. 7B). Both groups were, however, at chance for the first session of the FR16 and FR16-1 stages (*t <* 1). Neither group improved on the FR16 stage, so that their scores were still not above chance levels by the final, sixth session (RSC3: *t_11_* = −0.60, *p* = 0.56; Sham3: *t_12_* = −2, *p* = 0.07; Fig. 7B). The pattern was different, however, for the FR16-1 stage as both groups now appeared to discriminate the contingencies. This difference was most evident in the RSC3 group, which was above chance during the final session of the FR16-1 stage (*t_11_* = 4.03, *p* = 0.002; Fig. 7B). Meanwhile, whilst the Sham3 group’s performance was still at chance (Sham3: *t_12_* *=* 1.72, *p* = 0.112; Fig. 7B) they had greatly improved (from 47% HRwd choices at Session 1–68% at Session 6).

There was no group difference in the mean sessions to criterion for the FR8 stage (*t <* 1; Fig. 7A). Nor were there any Group differences, or Group by Session interactions, for the FR12, FR16 and FR16-1 stages (*F <* 1; Fig. 7B). There was no main effect of session during either the FR12 (*F*_2.3,52.8_ = 2.56 *p* = 0.08) or FR16 stages (*F*_1.9,44.6_ = 2.42, *p* = 0.1). Conversely, when the value of the LRwd lever was dropped from two pellets to one (i.e., the FR16-1 stage) there was a main effect of session (*F*_2.2,50.7_ = 14.21 *p <* 0.001), with the fraction of HRwd choices increasing from Session 1 to Session 6 (*F*_1,24_ = 15.04 *p <* 0.001),

## 5 Experiment 4: matching-to-place

No evidence was found from Experiment 3 that retrosplenial lesions disrupt the balance between reward size and cost. Next, a new cohort of operated rats was trained in a T-maze on a reinforced test of spatial working memory. The critical feature was that, following a baited sample run, rats were reinforced for returning to the *same* side arm of the T-maze. This matching-to-place behaviour is contrary to their innate bias to nonmatch [45], [46].

### 5.1 Methods

#### 5.1.1 Subjects

Experiment 4 involved a new cohort (RSC3) of male Lister hooded rats. Training started approximately 2 months post-surgery. Details of housing and husbandry are the same as the previous experiments. Prior to the current experiment, all rats completed a single appetitive operant task.

#### 5.1.2 Surgical procedures

The surgical procedures followed Cohort 2 (Experiments 2 and 3).

#### 5.1.3 Apparatus

**Fig. 8:**
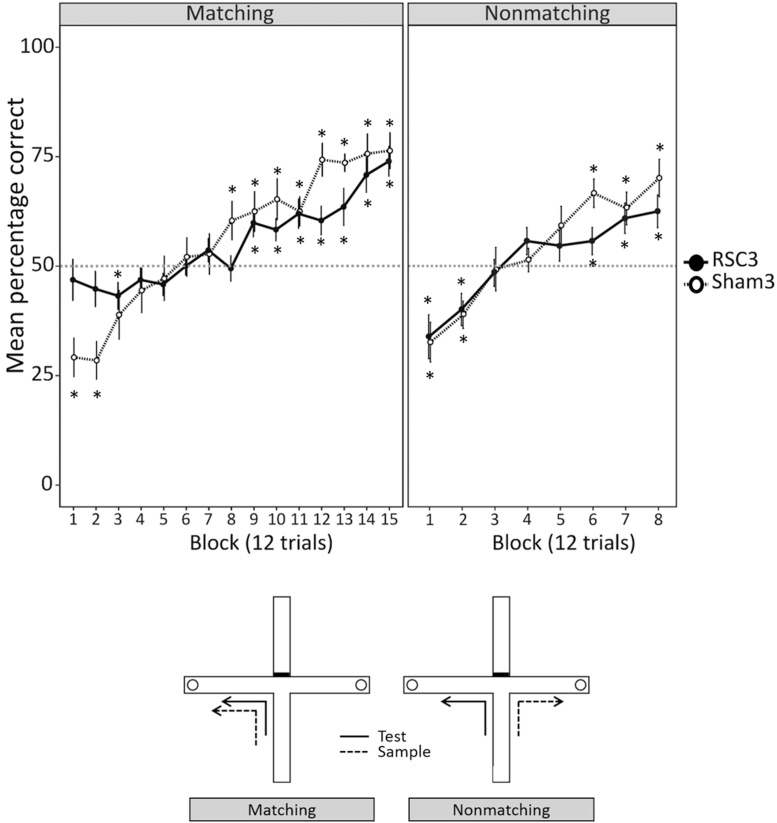
T-maze: matching and nonmatching (Experiment 4). The upper panels show the mean percentage correct scores across the matching (left panel) and nonmatching (right panel) tasks. The dotted line marks the chance performance level. Asterisks show when the performance of each group was significantly different from chance (*p <* 0.05; one sample *t*-test). Error bars show ±SEM. The lower panels depict the test protocols for the matching (left) and nonmatching (right) tasks in a T-maze. The circles correspond to food wells.

All testing took place in a modifiable cross-maze (Fig. 8). Each of the four arms was 70 cm long and 10 cm wide with wooden floor and clear Perspex walls (17 cm high). One of the arms was blocked off for the entire experiment to form a T-shaped maze. At the end of each cross arm there was a circular food well in which sucrose pellets (45 mg, Sandown Instruments, UK) were placed during training. The stem of the T-maze was designated as the start arm and remained so for the entire experiment. Consequently, the T-maze remained in the same orientation throughout. An aluminium barrier could be positioned ∼25 cm from the end of the start arm to create a start area. The maze, was elevated on a 94 cm high stand and was situated in a rectangular room (280 cm × 280 cm × 210 cm) that had salient visual cues attached to the walls.

#### 5.1.4 Behavioural training

##### 5.1.4.1 T-maze matching-to-place

Each session consisted of six trials and all animals completed one session a day. Each session consisted of three correct left and three correct right trials, presented in a pseudorandom order. Each trial comprised two stages, a ‘sample run’ followed by a ‘test run’. At the start of each trial, two sucrose pellets were placed in each food well and a metal barrier was placed at the choice point of the T-maze, thereby closing one cross arm (Fig. 8).

On a sample run, the animal was placed in the start area and the aluminium barrier removed, allowing the rat to run down the start arm. Because of the metal barrier blocking the entrance to one of the cross arms, the rat could only enter the one open arm. Once the rat had collected the sucrose pellets from the well at the end of the open arm, the rat was returned to the start area, where it remained for 10 s while the barrier at the choice point was removed and the same arm as previously visited was rebaited. The test run started as the start arm barrier was raised, allowing the animal a free choice between the two cross arms of the T-maze. The animal was deemed to have chosen an arm when it had placed a hindfoot within that arm; no retracing was allowed. If the rat had “matched”, i.e., had entered the arm previously visited on the sample run, it was allowed to eat the food reward and was then returned to the holding box. If the incorrect arm (i.e., the arm not previously visited on the sample run) was entered, the rat was allowed to run to the end of the arm and then returned to the holding box. The rats were tested in groups of three or four with each rat completing one trial before being returned to the holding box and waiting until all the other rats in the group had completed one trial, so that the inter-trial interval was typically between 3 and 4 min.

Initially, all rats completed 30 days (i.e., 180 trials) of matching training. These 30 sessions were followed by a five week break from T-maze training, during which the rats carried out no other tasks. All animals then received a further eight sessions of matching training, to ensure their performance was back up to criterion, before moving on to the nonmatching task.

##### 5.1.4.2 T-maze nonmatching-to-place

The structure of the nonmatching-to-place task was the same as the matching task, except that the rule was reversed, i.e., the correct test arm was the arm *not* visited on the sample run. All animals completed 16 sessions (i.e., 96 trials) of nonmatching training.

##### 5.1.4.3 Data analysis

For both the matching and nonmatching tasks, the total number of correct trials was calculated for blocks of 12 trials (i.e., two sessions). These scores were then analysed using a mixed ANOVA with the repeated measures factor Block and the between subjects factor Group.

In addition, for both the matching and nonmatching tasks, acquisition was divided into two phases: “perseveration” and “learning” [35]. The perseveration phase was defined as the period when rats were performing significantly below chance (≤3 out of 12, p = 0.073 binomial distribution). For the matching rule, this period corresponds to when rats were attempting to solve the matching task by relying on the innate instinct to alternate [47]. For the nonmatching task, this perseveration period reflects the continued use of the previously learnt matching strategy. The learning phase corresponded to when performance was at, or above, chance (i.e., when the rats had overcome the innate alternation bias [matching] or when they had successfully learnt the new strategy [nonmatching]). The two phases were defined by analysing the correct responses in a running window of 12 trials. Starting at trials 1–12 and advancing one trial at a time, the perseveration phase ended, and the learning phase began, at the point at which a rat achieved a score of ≥4 correct trials within a 12 trial window. Errors were normalised by the total number of trials in each phase (i.e., $\text{Error}\;\;\;\text{ fraction}=\frac{\text{Total}\;\;\text{ errors}\;\;\;\text{ in}\;\;\text{ phase}}{\text{Total}\;\;\;\text{ trials}\;\;\text{ in}\;\;\text{ phase}}$).

Finally, the degree of side bias displayed by each animal was defined for each session of the learning phase for both the matching and nonmatching tasks. The side bias was only calculated for those sessions in which all six of the trials were classified as learning trials; sessions containing a mix of perseveration and learning trials were excluded. A side bias session was defined as any session in which the rat turned in the same direction on ≥5 out of the 6 trials (i.e., ≥83%). The percentage of learning sessions in which an animal displayed a side bias was then compared between the two groups using a paired *t*-test.

### 5.2 Results

#### 5.2.1 Histology

All of the 16 RSC3 rats displayed considerable cell loss in both the granular and dysgranular subregions. In one rat there was, however, bilateral sparing of granular retrosplenial cortex rostral to the splenium. A further six animals had minimal sparing of granular A cortex caudal to the splenium. One of these six also had unilateral sparing in the most rostral part of retrosplenial cortex. Of the 16 RSC3 animals, nine had a limited degree of bilateral hippocampal damage (Fig. 2). The extent of this damage varied across animals but was confined within dorsal CA1 in all cases. In five of these animals this bilateral damage affected mainly the medial edge of dorsal CA1, i.e., distal CA1. The remaining four animals had even more restricted bilateral damage within the same area. As is sometimes seen following lesions in the retrosplenial cortex, around half of the RSC3 group had ventricular dilatation. In 10 of the 16 animals, there was some minor damage to anterior cingulate cortex restricted to the border with retrosplenial cortex. Following histological analyses the final group sizes were; RSC3 n = 16, Sham3, n = 12.

#### 5.2.2 Behaviour

##### 5.2.2.1 Error analysis

The performance of RSC3 group was at chance at Block 1 and 2 and from Block 4 to Block 8 (all *p >* 0.05; one sample *t* test; see Supplementary Table 2). From Block 9 to Block 15 their performance was above chance (all *p <* 0.05; one sample *t* test; see Supplementary Table 2). Conversely, the performance of the Sham3 group was below chance for the first two blocks (all *p <* 0.05; one sample *t* test; see Supplementary Table 2) and above chance from Block 8 to Block 15 (all *p <* 0.05; one sample *t* test; see Supplementary Table 2 and Fig. 8).

When the percentage of correct matching trials was compared between the two groups using blocks of 12 trials there was no main effect of Group (*F <* 1; Fig. 8). However, there was a significant Group by Block interaction (*F*_6.2161_ = 3.04, *p* = 0.007). Simple effects revealed that the RSC3 group made significantly more correct responses during the first (*F*_1,26_ = 7.14, *p* = 0.01) and second (*F*_1,26_ = 7.54, *p* = 0.01) blocks. This pattern was reversed in later blocks; at Block 8 (*F*_1,26_ = 4.71, *p* = 0.04) and Block 12 (*F*_1,26_ = 7.75, *p* = 0.01) the Sham3 group made more correct responses than the RSC3 group. By the final block (Block 15), the performance of both groups was comparable (*F <* 1; Fig. 8).

For the nonmatching stage, the performance of RSC3 group was below chance for the first two blocks (*p <* 0.05; one sample *t* test). For the final two blocks, their performance was above chance (*p <* 0.05; one sample *t* test). The performance of the Sham3 group was also below chance for the first two blocks (*p <* 0.05; one sample *t* test) and they performed above chance for the final three blocks (*p <* 0.05; one sample *t* test; Fig. 8). There was no main effect of Group (*F <* 1) or Group by Block interaction (*F*_7182_ = 1.1, *p* = 0.36) on the percentage of correct trials during the nonmatching task (Fig. 8).

##### 5.2.2.2 Perseveration vs. learning phase

**Fig. 9:**
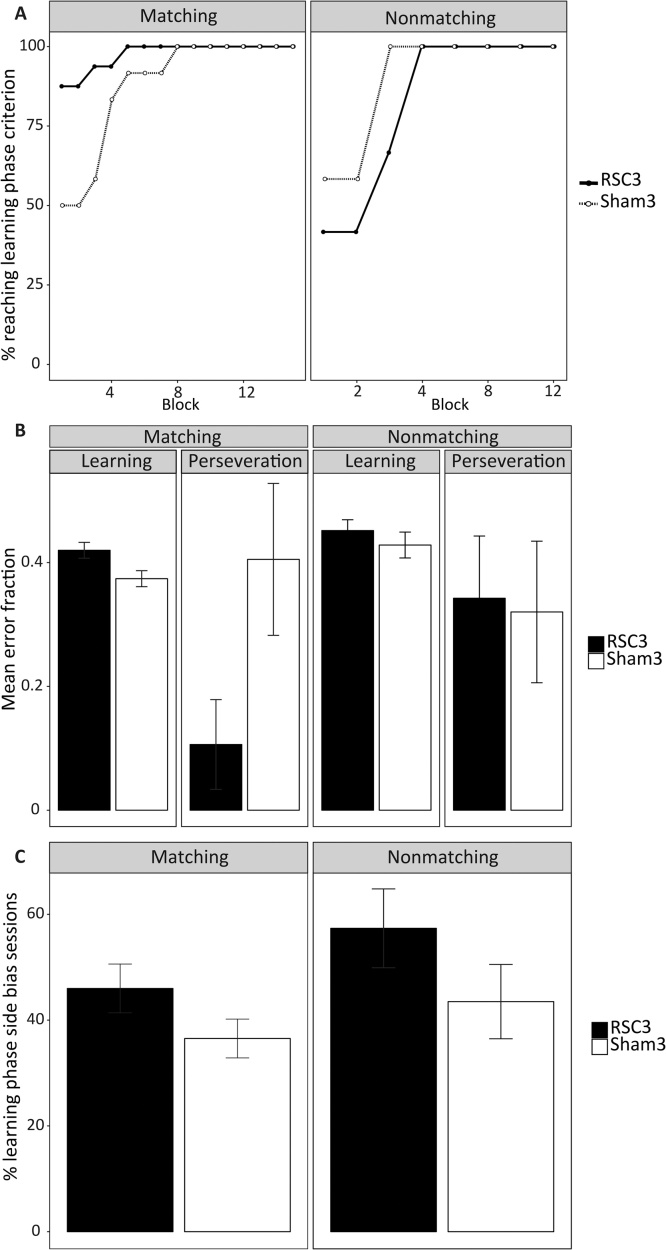
T-maze: matching and nonmatching (Experiment 4). (A) The percentage of rats reaching the learning phase (i.e., ≥4 correct trials out of 12) at each block for the matching (left panel) and nonmatching (right panel) tasks. (B) The mean fraction of errors made during the learning and perseveration phases for the matching and nonmatching tasks. Errors were normalised by the total number of trials in each phase. (C) The mean percentage of learning sessions in which animals displayed a side bias for the matching and nonmatching tasks. A side bias session was defined as any session in which the rat turned in the same direction on ≥5 out of the 6 trials (i.e., ≥83%). The side bias was only calculated for those sessions in which all six of the trials were classified as learning trials; sessions containing a mix of perseveration and learning trials were excluded. Error bars show ±SEM.

Fig. 9A shows the percentage of animals in each group that had reached the criterion for the learning phase at each block (i.e., when performance was at or above chance). For the matching task, the RSC3 group reached the learning phase earlier on in training than did the Sham3 group. For the nonmatching task the reverse was true, with the Sham3 group reaching the learning before the RSC3 group.

The fraction of errors (see Methods) in each phase was compared between the groups. For the matching task, there was no overall difference in the fraction of errors between the learning and perseveration phases (*F*_1,26_ = 3.76, *p* = 0.06; Fig. 9C). There was, however, a significant Group by Phase interaction (*F*_1,26_ = 5.60, *p* = 0.03), with the RSC3 group making significantly more learning phase errors than the Sham3 group (*F*_1,26_ = 6.10, *p* = 0.02). Conversely, the Sham3 group made significantly fewer perseveration phase errors than the RSC3 group (*F*_1,26_ = 4.91, *p* = 0.04).

For the nonmatching task there was no difference between the groups in the fraction of errors made during either phase (*F <* 1). Nor was there an overall difference in the fraction of errors between the learning and perseveration phase (*F*_1,26_ = 2.02, *p* = 0.17).

##### 5.2.2.3 Side Bias

For the matching task, there was no difference between the groups in the percentage of learning phase sessions in which animals displayed a side bias (i.e. ≥ 83% test trials in same direction; *t*_26_ = 1.52, *p* = 0.14; Fig. 8D). Similarly, for the nonmatching task, there was no difference between the groups in the percentage of learning phase sessions in which animals displayed a side bias (*t*_26_ = 1.31, *p* = 0.20; Fig. 9C).

#### 5.2.3 Impact of incidental hippocampal damage (Experiments 1–4)

Of the 16 RSC3 rats, nine had evidence of some bilateral hippocampal damage (see above). Therefore, the lesion group was split according to whether there was bilateral hippocampal damage (subgroup RSC3-BiHpc, n = 9) or not (subgroup RSC3-UniHpc, n = 7).

A mixed ANOVA, with the between subjects factor Group (RSC3-BiHpc and RSC3-UniHpc) and the within subject factor Block, was conducted on the percentage of correct responses. For the matching task, there were no effects of Group or interaction between Group and Block (both *F <* 1). Again, for the nonmatching task, there was no main effect of group (*F <* 1). Curiously, a Group by Block interaction (*F*_7,98_ = 2.35, *p* = 0.03) arose because the RSCBiHpc group made fewer errors at block five than the RSC3-UniHpc group (simple effects *F*_1,15_ = 5.4, *p* = 0.04), i.e., those rats with bilateral, additional damage briefly outperformed those with unilateral damage.

## 6 General discussion

There is both anatomical and functional evidence to suggest that the rodent retrosplenial cortex might have an important role in supporting frontal functions. Anatomically, retrosplenial cortex is directly linked with the anterior cingulate cortex and indirectly linked with prelimbic cortex, via the thalamus and the anterior cingulate cortex [16], [17], [19]. Previous lesion studies have shown that, in addition to disrupting tests of spatial memory [4], [11], [48], retrosplenial cortex lesions can impair recency memory (Powell et al. [22]), disrupt a rodent analogue of the Stroop task [20], and impair crossmodal object recognition [26]. All of these nonspatial tasks are closely associated with frontal cortex function in rodents [21], [23], [24], [25], [27], pointing to joint functional contributions. To assess the likelihood that the rat retrosplenial cortex has a more general role in supporting frontal functions, the present study employed three tasks (Experiments 1, 2, 4) that are sensitive to prelimbic cortex lesions (extradimensional shifts in a digging task, strategy shifting in an automated apparatus, and matching-to-place in a T-maze). In addition, both matching-to-place and cost-benefit discrimination tasks (Experiment 3) are sensitive to anterior cingulate damage [33], [34], [35], as well as intradimensional set learning [29].

Despite prior evidence that retrosplenial cortex might closely support frontal functions, there was no evidence for this prediction from the present pattern of results. Performance on the intradimensional/extradimensional shift task (Experiment 1) and the cost-benefit discrimination (Experiment 3) appeared unaffected by the surgeries. The only lesion effect on the strategy-shifting task (Experiment 2) was manifested as an enhanced rate of initial shifting from the visual to the response-based task, i.e., opposite to that associated with prelimbic inactivation [30]. Finally, although the retrosplenial lesions affected performance on the matching-to-place task, again the pattern of errors did not reflect that seen after prelimbic cortex lesions [35].

A possible concern is that the null results in Experiments 1–3 might stem from retrosplenial tissue sparing. This possibility seems, however, unlikely. As explained, two different cohorts of rats with retrosplenial cortex lesions, along with their sham controls, were tested in these particular experiments. In both cohorts, the retrosplenial surgeries involved tissue along almost the entire length of the region, a potentially important factor as lesions of more limited length can have null effects (Neave et al. [43]; Aggleton & Vann [49]). In addition, Cohort 1 was separately found to be impaired on tests of crossmodal recognition and spatial memory [26], [50], while Cohort 2 was impaired on tests of object recency memory [22].

In the case of Cohort 3 (matching-to-place, Experiment 4), a different concern is whether the behavioural impairments arose from the cortical lesions encroaching into other areas, most notably the hippocampus. For this reason, those cases with unintended bilateral hippocampal damage were compared with the remaining RSC3 rats. While some unilateral hippocampal cell loss was typically seen in these remaining cases, it is known that even very extensive, unilateral hippocampal damage can spare T-maze nonmatching-to-place [51]. Critically, there was no evidence that the bilateral hippocampal encroachment had effects over and above that associated with retrosplenial cortex damage on either matching or nonmatching in the present study.

The digging task used in Experiment 1, which sequentially taxes intradimensional and extradimensional-shifts, was of much interest as previous studies have provided a double dissociation between the effects of lesions involving the prelimbic cortex [28] and the anterior thalamic nuclei [38]. While prelimbic lesions impair extradimensional shifts [28], i.e., the ability to switch from one domain of cues to another, lesions of the anterior thalamic nuclei produce the opposite pattern, impairing the ability to focus within a particular class of cues (intradimensional shift) while enhancing extradimensional shifts [38]. Anterior cingulate lesions may also affect intradimensional shifts [29]. The anterior thalamic lesion effects are of particular interest as retrosplenial cortex has an especially close affinity with these thalamic nuclei [4]. This affinity is seen in their dense interconnections [3], [40], [41], [52], [53], the common effects of lesions in both sites on spatial tasks [7], [54] and the ways in which anterior thalamic damage disrupts retrosplenial cortex activity and plasticity [55], [56], [57].

For these reasons, it was striking that the rats with retrosplenial lesions in the present study showed no disruption to intradimensional or extradimensional shift behaviour. These null results appear to contrast with those from a previous study that also examined a series of digging-based discriminations, in which it was reported that small retrosplenial cortex lesions impair intradimensional shifts [29]. It may, however, be significant that the behavioural task [29] was different from that used in the current study as it involved fewer discriminations in which to establish an intradimensional shift. This difference may explain why, in that study [29] there was no behavioural evidence of an attentional set having been acquired by the control group. For this reason, we used a protocol [37] with more initial discriminations to better ensure the formation of an attentional set. The present null results suggest that the role of the anterior thalamic nuclei in intradimensional shifts is more closely linked to their frontal, rather than their retrosplenial, connections. The contrasting effects of prelimbic and anterior thalamic damage potentially reflect complementary aspects of attention [58].

The question of whether retrosplenial lesions affect the ability to switch strategies was further explored in Experiment 2. Here, the task involved changing from a discrimination based on visual stimuli to one based on response position (left or right). The retrosplenial lesions did not affect the ‘cost’ of switching. This result can be contrasted with the switching deficits seen in rats with temporary lesions in medial prefrontal cortex [30]. This contrast is highlighted by the way in which the present rats with retrosplenial lesions showed accelerated switching from a visual to a response-based discrimination, a direction of effect diametrically opposed to that seen after inactivation of medial frontal cortex [30]. Using other spatial-visual strategy shifts it has again been found that medial frontal inactivation impairs switching, while anterior cingulate inactivation can have no apparent effect [31]. The latter result is more closely allied to the present outcome of retrosplenial lesions.

It is striking that, after switching from the response to the visual-guided discrimination, nearly all of the errors made by both groups (RSC2 and Sham2) were “perseverative” (i.e. the lever pressed was the one which had been rewarded on the previous response discrimination stage). Clearly, all animals found it very difficult to update a previously learnt spatial discrimination.

Experiment 4, matching-to-place in a T-maze, again looked at the ability to switch strategies. Here, the shift was from a spontaneous strategy (nonmatching) to a reinforced strategy (matching), followed by a switch back to nonmatching (now reinforced). Error analysis showed that the retrosplenial lesion impairment on the matching task was not, however, due to strategy perseveration, as is the case after frontal lesions [35]. Rather, the retrosplenial lesions appeared to shift the baseline of performance so that the scores moved closer to chance (Fig. 8). This shift meant that, when compared to their controls, the lesioned rats had superior scores for the initial sessions but inferior scores over the final matching sessions.

Taken together, the effects seen in rats with lesions in the retrosplenial cortex in both Experiment 2 (facilitated acquisition of a spatial discrimination) and Experiment 4 (a weaker alternation bias) are consistent with a short-term spatial memory deficit. Since the levers in the operant chambers could only be approached from one direction, the response-based discrimination in Experiment 2 may have been solved using either allocentric or egocentric spatial representations. Therefore, it is possible that the retrosplenial lesioned animals showed facilitated acquisition of this discrimination strategy due to a reduced competition between spatial representations. Indeed, retrosplenial cortex has been implicated in mediating between different spatial reference frames [59].

Similarly, the simplest explanation for the pattern of results seen in the matching stage of Experiment 4 is that the lesions led to a mild spatial memory deficit. As a consequence, although the rats with retrosplenial lesions spontaneously nonmatched at the start of training (i.e., they initially applied the incorrect rule, resulting in scores below chance), their spatial working memory errors raised their scores above those of the controls, who nonmatched more accurately. For the same reason, this spatial memory deficit would also be expected to reduce scores, relative to controls, once the matching rule was learnt. Evidence that comparable retrosplenial cortex lesions have mild effects, most evident at the outset of T-maze alternation training [13], [14], would appear to support this account. At the same time, these results highlight qualitative differences between the impact of retrosplenial lesions and lesions in the hippocampus, anterior thalamic nuclei, and prelimbic cortex [35], [51].

Experiment 3 had a slightly different goal to the other studies as it was not focussed on strategy switching. Rather, the experiment examined the choice between low reward/low effort and high reward/high effort. This task was selected as it is sensitive to anterior cingulate cortex damage [34]. The effectiveness of the present task can be seen, for example, in the final stage where both the control rats and those with retrosplenial lesions switched their choice behaviour when the low reward lever gave one rather than two pellets (FR16-1, Fig. 7). There was, however, no differential effect of retrosplenial cortex tissue loss. This pattern of dissociable effects between anterior cingulate and retrosplenial cortex damage is seen in other studies [60].

Taken together, the current results suggest that the retrosplenial cortex is not required for tracking predictive relationships between stimuli and rewards, e.g., updating behaviour when environmental contingencies change or inhibiting a previously reinforced response. This pattern of results is striking because, in the spatial domain, retrosplenial lesion effects often only emerge when animals are required to switch between different spatial strategies [4], [14], [15]. The current set of null results suggests that, in the non-spatial domain at least, this ability to switch between different cue and strategy types need not depend on retrosplenial cortex.

Our understanding of retrosplenial cortex function often emphasises its close connections with the hippocampal formation, the parahippocampal region, and the anterior thalamic nuclei [4], [61], [62], [63]. The present study examined whether the retrosplenial cortex might also provide an interface for an additional set of direct connections, namely those with the anterior cingulate cortex, as well as indirect connections with other frontal areas. This possibility was tested using a variety of spatial and nonspatial tasks. The overall pattern is complex as retrosplenial cortex lesions can disrupt some frontal tasks (e.g., a ‘Stroop’ task analogue, recency memory discriminations, crossmodal object recognition) yet spare others e.g., intra and extradimensional shifts, reversal learning, as well as automated delayed nonmatching-to-position [43], [64]. One common factor that differentiates those tasks which reveal a retrosplenial deficit from those that do not, is the degree to which animals directly experience changes in reward contingencies. In the current set of experiments, such changes occurred directly ‘on-line’ and consequently the animals were able to update responding accordingly. Conversely retrosplenial lesion deficits emerge on non-spatial tasks when animals have to rely on previously acquired representations to solve the current problem or switch between different representations of the same event [12], [20], [22], [65], [66].
