# Supplementary material for: The rat retrosplenial cortex as a link for frontal functions: A lesion analysis
Source: Behav Brain Res. 2017 Sep 29;335:88–102. doi: 10.1016/j.bbr.2017.08.010 (PMC5597037; doi:10.1016/j.bbr.2017.08.010)
Supplement: Supplementary file 1 [file mmc1.docx]

| Lesion | SD | CD | ID1 | ID2 | ID3 | ID4 | ED | Rev |
| --- | --- | --- | --- | --- | --- | --- | --- | --- |
| RSC1 | 12.77 (SE±0.68) | 9.77  (SE±0.75) | 12.77  (SE±0.93) | 12.08  (SE±0.89) | 9.69  (SE±0.68) | 8.08  (SE±0.37) | 15.62  (SE±0.62) | 11.69  (SE±0.52) |
| Sham1 | 13.67  (SE±0.48) | 11.00  (SE±0.58) | 13.83  (SE±0.42) | 12.67  (SE±0.66) | 10.83  (SE±0.68) | 8.33  (SE±0.22) | 15.75  (SE±0.54) | 12.33  (SE±0.69) |

Table 1. Mean trials to criteria for each group at each discrimination stage. SD = simple discrimination, CD = complex discrimination, ID = intradimensional discrimination, ED = extradimensional discrimination, Rev = Reversal.
