# Supplementary material for: The rat retrosplenial cortex as a link for frontal functions: A lesion analysis
Source: Behav Brain Res. 2017 Sep 29;335:88–102. doi: 10.1016/j.bbr.2017.08.010 (PMC5597037; doi:10.1016/j.bbr.2017.08.010)
Supplement: Supplementary file 2 [file mmc2.docx]

| Block | Group | Stage | *t*-statistic | *p*-value | df | Chance |
| --- | --- | --- | --- | --- | --- | --- |
| 1 | RSC3 | Matching | -0.67 | 0.515 | 15 | = |
| 1 | Sham3 | Matching | -4.72 | 0.001 | 11 | < |
| 2 | RSC3 | Matching | -1.30 | 0.214 | 15 | = |
| 2 | Sham3 | Matching | -5.02 | <0.001 | 11 | < |
| 3 | RSC3 | Matching | -2.21 | 0.043 | 15 | < |
| 3 | Sham3 | Matching | -2.00 | 0.071 | 11 | = |
| 4 | RSC3 | Matching | -1.14 | 0.270 | 15 | = |
| 4 | Sham3 | Matching | -1.10 | 0.296 | 11 | = |
| 5 | RSC3 | Matching | -1.65 | 0.119 | 15 | = |
| 5 | Sham3 | Matching | -0.55 | 0.594 | 11 | = |
| 6 | RSC3 | Matching | 0.00 | 1.000 | 15 | = |
| 6 | Sham3 | Matching | 0.48 | 0.643 | 11 | = |
| 7 | RSC3 | Matching | 1.39 | 0.186 | 15 | = |
| 7 | Sham3 | Matching | 0.60 | 0.560 | 11 | = |
| 8 | RSC3 | Matching | -0.18 | 0.860 | 15 | = |
| 8 | Sham3 | Matching | 2.39 | 0.036 | 11 | > |
| 9 | RSC3 | Matching | 3.13 | 0.007 | 15 | > |
| 9 | Sham3 | Matching | 2.76 | 0.019 | 11 | **>** |
| 10 | RSC3 | Matching | 3.30 | 0.005 | 15 | > |
| 10 | Sham3 | Matching | 3.26 | 0.008 | 11 | **>** |
| 11 | RSC3 | Matching | 3.62 | 0.003 | 15 | > |
| 11 | Sham3 | Matching | 3.76 | 0.003 | 11 | **>** |
| 12 | RSC3 | Matching | 3.18 | 0.006 | 15 | > |
| 12 | Sham3 | Matching | 6.46 | <0.001 | 11 | **>** |
| 13 | RSC3 | Matching | 3.20 | 0.006 | 15 | > |
| 13 | Sham3 | Matching | 11.76 | <0.001 | 11 | **>** |
| 14 | RSC3 | Matching | 5.27 | <0.001 | 15 | > |
| 14 | Sham3 | Matching | 5.68 | <0.001 | 11 | **>** |
| 15 | RSC3 | Matching | 7.06 | <0.001 | 15 | > |
| 15 | Sham3 | Matching | 6.47 | <0.001 | 11 | **>** |
| 1 | RSC3 | Non-matching | -3.22 | 0.006 | 15 | **<** |
| 1 | Sham3 | Non-matching | -3.84 | 0.003 | 11 | **<** |
| 2 | RSC3 | Non-matching | -2.70 | 0.016 | 15 | **<** |
| 2 | Sham3 | Non-matching | -3.55 | 0.005 | 11 | **<** |
| 3 | RSC3 | Non-matching | -0.51 | 0.617 | 15 | **=** |
| 3 | Sham3 | Non-matching | -0.14 | 0.891 | 11 | **=** |
| 4 | RSC3 | Non-matching | 1.84 | 0.085 | 15 | **=** |
| 4 | Sham3 | Non-matching | 0.52 | 0.615 | 11 | **=** |
| 5 | RSC3 | Non-matching | 1.31 | 0.208 | 15 | **=** |
| 5 | Sham3 | Non-matching | 1.95 | 0.078 | 11 | **=** |
| 6 | RSC3 | Non-matching | 1.84 | 0.085 | 15 | **=** |
| 6 | Sham3 | Non-matching | 5.14 | <0.001 | 11 | **>** |
| 7 | RSC3 | Non-matching | 3.16 | 0.006 | 15 | **>** |
| 7 | Sham3 | Non-matching | 3.51 | 0.005 | 11 | **>** |
| 8 | RSC3 | Non-matching | 3.35 | 0.004 | 15 | **>** |
| 8 | Sham3 | Non-matching | 4.70 | 0.001 | 11 | **>** |

Table 2. Summary of *t*-statistics and *p*-values for the one sample t-tests comparing group performance at each block of the matching and non-matching to chance performance (i.e. 50%). The final column shows whether performance was below (<), above (>) or not different from chance (=). Df = Degrees of freedom.
